# Editorial of Special Issue “Multiple Sclerosis: Diagnosis and Treatment II”

**DOI:** 10.3390/biomedicines9111605

**Published:** 2021-11-03

**Authors:** Victor M. Rivera

**Affiliations:** Department of Neurology, Baylor College of Medicine, One Baylor Plaza, Houston, TX 77030, USA; vrivera@bcm.edu; Tel.: +1-832-407-0668

**Keywords:** multiple sclerosis, acetylcholine, PBMCs, minority populations, spinal cord, sphingosine-1-phosphate modulators, neurofilaments light chain, biomarkers, microRNAs, child neurology

## Abstract

The special issue on Multiple Sclerosis: Diagnosis and Treatment II, reflects advances and discoveries in the molecular and cellular mechanisms of disease, and novel laboratory techniques providing more sensitivity to diagnostic techninques and the understanding of neuroinflammation. Mitochondrial-mediated apoptosis in isolated peripheral blood mononuclear cells and the role of reactive oxygen species are studied as indicators of activity of MS. In these cells, downregulation of circular and linera RNAs are reported as markers of highly active disease in MS. Progress and importance of Neurofilaments determinations in early diagnosis and as a marker of disease activity, and the analysis of the complex mechanisms and therapeutic potential of Sphingosine-1-phosphate receptor modulator are discussed. Epidemiologic observations from a highly diversified area of the world provide more insights into this important aspect of MS; discussions on the clinical challenges posed by spinal cord involvement in demyelinatind disorders and the latest aspects of pediatric onset MS, complement this fine collection of scientific papers.

Evolution in the knowledge of previously obscure molecular mechanisms underlying clinical manifestations of multiple sclerosis (MS) has been truly remarkable and has added to the progress of diverse branches of neurosciences. Understanding new molecular (beyond some of the known immunologic mechanisms) and biochemical aspects contributing to the intimate mechanism of the disease, while definitely adding to the complexity of MS, concomitantly provides the potential to translate alterations of these paths into therapeutic interventions for this multifaceted neurological disorder.

Epidemiologically, MS is the most common inflammatory, demyelinating, and degenerative disease of the central nervous system (CNS). In the US, Canada, the British Isles, Scandinavia, Northern Europe, and other areas of the world where MS has a considerable prevalence, this condition constitutes the most common cause of neurological disability in young adults after head injury. In addition, current data indicate the presence of MS in practically every ethnic group in the world.

Etiologically, MS represents a multifactorial complex whereby numerous environmental elements with epigenetic effects contribute to triggering the disease in a genetically susceptible individual.

Mechanistically, MS is an autoimmune disease, with its typical erroneous immune cascade initiating by activation of peripheral T-cell lymphocytes by antigen-presenting cells. Under influences from B-cell lymphocytes and other molecular stimulations, cell division increases, and the permeability of the blood–brain barrier is favored by the release of pro-inflammatory cytokines by T-cells. This setting facilitates the trafficking of immune cells into the CNS with the consequent attack of myelin and eventually axonal injury. A gamut of clinical neurological manifestations will result from damage to these CNS structures.

Many of the molecular mechanisms involved in this process are unknown. Acetylcholine (Ach) participates in the modulation of central and peripheral inflammation irrespective of the causation, ergo in neuroinflammation in MS. T and B cells, macrophages, dendritic cells, microglia, astrocytes, and oligodendrocytes all play a consequential role in the immunology involved in the disease, and all express cholinergic markers and receptors of muscarinic and nicotinic type. The Ach hydrolyzing enzymes acetylcholinesterase and butyrylcholinesterase are reportedly reduced in MS while (not necessarily in a homeostatic response) pro-inflammatory cytokines (IL-6, Tumor Necrosis Factor, and interferon-gamma among others) are released in excess by activated T-cells. Genetic influences are shown by the influence of polymorphisms in the enzymatic activity, and the expression of cholinergic markers in serum and cerebrospinal fluid (CSF) during clinical exacerbation in patients with relapsing disease, and in the chronic phases of the animal model with experimental autoimmune encephalomyelitis (EAE), demonstrate the possible correlation between cholinergic system alterations and neuroinflammation in MS [1].

Since MS pathophysiology largely depends on cellular behavior, the proliferation and increased division of activated lymphocytes are confounded when mitochondria-mediated apoptosis is impaired. Several mechanisms and proteins—including optic atrophy 1 (OPA1) protein, PHB2 protein, and increased reactive oxygen species (ROS)—regulate mitochondrial dynamics and release pro-apoptotic factors. These phenomena were studied utilizing sophisticated techniques in isolated peripheral blood mononuclear cells (PBMCs), demonstrating deregulation of OPA1 processing and increased PHB2 and ROS. As postulated by the investigators, these molecular parameters could also be useful to evaluate MS activity [2]. In addition, increased ROS and its role in the senescence of the immune system are being studied at present.

Epidemiological observations have shown that in addition to global differences in MS prevalence among ethnic and racial groups, disease severity and clinical features appear to differ among populations. A retrospective analysis performed on multi-ethnic cohorts in Houston, Texas, US, disclosed substantial disparities in epidemiological distributions, risk factors, neurological disability, and magnetic resonance imaging (MRI) findings among the groups [3]. Houston is reportedly the most ethnically diverse city in the US. In this study, African Americans (AA) and Hispanics (people of Latin American extraction) had a lower incidence than White patients but had greater disease encumbrance and disability in earlier stages of the disease. At the onset, however, mean age and degree of brain atrophy were similar, as was the degree of volume loss progression over time and the burden of T1, T2, and gadolinium-enhancing lesions. The study showed that active smokers had significantly increased odds of greater disability both at diagnosis and at last clinical encounter compared to nonsmokers (OR: 2.44, 95% CI: 1.10–7.10, OR = 2.44, 95% CI: 1.35–6.12, *p =* 0.01, respectively).

This study also disclosed the importance of access to care. Patients who were evaluated by a neurologist at symptom onset had significantly decreased disability (defined by the investigators as Expanded Disability Status Scare (EDSS) > 4.5) at last presentation compared to patients who were not evaluated by a neurologist (OR: 0.04, 95% CI: 0.16–0.9).

The accurate diagnosis of clinically definite MS continues to be a challenging task, involving a great responsibility for the practitioner since the current paradigm of “*early diagnosis, early treatment*” will reflect an improved prognosis. The internationally used McDonald Criteria 2017 version [4] is an essential diagnostic tool designed to amalgamate the clinical presentation (first event) along with specific MR imaging criteria and CSF analysis demonstrating the presence of Oligoclonal Bands. The conjunction of all these elements provides the cardinal principles of lesions *disseminated in space* and *disseminated in time*, and that is not a *better explanation* for the clinical picture. The proper utilization of the McDonald Criteria considerably reduces the possibilities of misdiagnosis as well.

Despite the progress accomplished in MS diagnostic identification, disease biomarkers have been notably lacking. Blood neurofilament light chain (NfL), neuronal-specific heteropolymers, have been established as a marker of neuronal and axonal injury in diverse neurodegenerative and inflammatory disorders. NfLs are light (low-molecular-weight) chains with a specific carboxy-terminal domain. Following synthesis and assembly in the neuron cell body, tetrameters of NfL proteins are transported bidirectionally along axons by the microtubular apparatus, forming an overlapping array that runs parallel to axons. Once formed, however, in a healthy state, they remain stable for months to years. In the presence of neuronal–axonal disease, NfL is released into the CSF and blood compartments, becoming specific indicators of CNS structural damage. NfL is reported to be significantly elevated in amyotrophic lateral sclerosis, Alzheimer’s disease, frontotemporal dementia, and other CNS-degenerative conditions, and even in some peripheral neuropathies. NfL was initially studied in CSF, but the invasive nature of the lumbar puncture procedure limited its utilization. More recently, the advent of ultrasensitive digital immunoassay technologies has enabled reliable detection and measurement in serum/plasma. A comprehensive review of the diagnostic potential of NfL in MS, and the association with clinical outcomes, emphasizes the next steps to overcome before this test is adopted on a routine clinical basis [5].

The presence of numerous clinical phenotypes and the development of a monophasic course (the majority of cases exhibit a relapsing/remitting course) are special challenges to consider in the differential diagnosis of MS, particularly in cases where the spinal cord is involved.

An actualized review of clinical aspects, MRI spinal cord lesion patterns, CSF profiles, and autoantibodies in conditions such as neuromyelitis optica (anti-Aquaporin-4 IgG), anti-Myelin oligodendrocyte glycoprotein (MOG), and anti-glial fibrillary acidic protein IgG-associated diseases underlines the understanding of individual case etiology to make adequate therapeutic decisions, crucial for the prognosis and long-term outcomes in patients with MS or its mimickers [6].

Treatment of MS has experienced remarkable progress since the advent of disease-modifying therapies (DMT) as injectable interferons and glatiramer acetate in the decade of 1990, considered at present as first-line or platform treatments. Pharmacological agents of more recent approval include some monoclonal antibodies (MABs) with specific molecular targets; three of these agents are administered by periodic intravenous infusion, and one is injected subcutaneously. An important addition to the therapeutic armamentarium constituted the emergence of oral medications: sphingosine-1-phosphate receptor (S1PR) modulators, fumarates, and cladribine. MABs and oral medications are rated in general in the range of moderate-to-high efficacy agents. The US Food and Drug Administration approved the first S1PR modulator, fingolimod, in 2010, and the European Medicine Agency approved in the following year.

The molecule sphingosine-1-phosphate, via its G-protein-coupled receptors, signals lymphocytes to egress from peripheral lymphoid organs. S1PR-antagonist agents promote sequestration of lymphocytes in the lymph glands and hence reducing lymphocyte-driven inflammatory damage of the CNS. Five S1P receptors have been identified (S1PR1-5). In 2020–2021, other S1PR-modulator drugs became available: Siponimod, Ozanimod, and Posenimod.

These agents reduce relapse risk, sustained disability progression, MRI markers of disease activity, and whole-brain atrophy. A review [7] of the molecular characteristics of this family of therapeutic modulators addresses the possibility of the development of more selective and intracellular S1PR-driven downstream pathway modulators for MS.

While the role of NfL as a diagnostic marker for MS is being considered (see above), an exploratory observational study assessed serum levels behaviors measured longitudinally before and after 24 months of treatment with first-line immunotherapy, carried out in patients with relapsing–remitting MS [8]. The medications utilized were the traditional platform therapies: interferon-beta and glatiramer acetate. Overall serum NfL was higher at time points concurrent with relapse than during remission periods (12.8 pg/mL vs. 9.7 pg/mL, *p =* 0.011). At follow-up, relapse-free patients showed significantly reduced serum NfL starting from 9 months compared to baseline (*p =* 0.05) and reduced levels after 12 months compared to baseline (*p =* 0.013) in patients without EDSS progression for 12 months. These data suggest that longitudinal measurements of serum NfL to monitor disease activity and therapy response in MS are one more potential aspect to explore for this biomarker.

Observations in MS patients with highly active disease (defined as a clinical course with frequent and repeated relapses and new T2 or gadolinium-enhancing lesions by MRI) show the presence of anti-myelin lipid-specific oligoclonal IgM bands (LS-OCMBs) in CSF to be an accurate predictor of an aggressive evolution of the disease. A disadvantage of this assessment is the need for an invasive spinal tap procedure. Investigators have studied the expression profile of circular RNA and linear RNA arrays in PBMCs from patients with LS-OCMBs. Two circular (hsa_circ_0000478 and hsa_circ_0116639) and two linear RNAs (*IRF5* and *MTRNR2L8*) were downregulated in PBMCs from patients with positive CSF bands (70% accuracy). The investigators propose that RNAs’ expression in peripheral blood cells might serve as minimally invasive biomarkers of highly active MS [9].

The clinical, laboratory, and experimental studies in this Special Issue address novel immunologic and molecular findings and their impact and influence on clinical manifestations in adult patients with MS. It is estimated that between 3 and 5% of all patients have an onset of disease under de age of 18 (considered as the pediatric age), and although MS is rare in children, it carries a significant physical and cognitive disability in these groups. Progress in the understanding of MS in children and the availability of pediatric MS diagnostic criteria is essential in differentiating this disorder from a myriad of complex neurological diseases affecting children. The incidence between males and females diagnosed before puberty is relatively equivalent. In adolescents, the ratio of females to males with MS increases to 2 to 3:1, similar to the gender distribution in adults. A review study [10] emphasizes the development of the International Pediatric MS Study Group in 2005 as a milestone in the progress of the knowledge base surrounding pediatric MS, including clinical manifestations and possibilities of treatment.

Knowledge of MS over recent decades has flourished substantially, resulting in notable scientific findings, the development of sensitive laboratory technologies, and enrichment of the understanding of the clinical aspects of this complex neurological disorder.

## Data Availability

Exclude.
